# Supplementary material for: Enhanced Hepatogenic Differentiation of Human Wharton’s Jelly–Derived Mesenchymal Stem Cells by Using Three-Step Protocol
Source: Int J Mol Sci. 2019 Jun 20;20(12):3016. doi: 10.3390/ijms20123016 (PMC6627410; doi:10.3390/ijms20123016)
Supplement: Supplementary file 1 [file ijms-20-03016-s001.pdf]

International Journal of
Molecular Sciences

MDPI

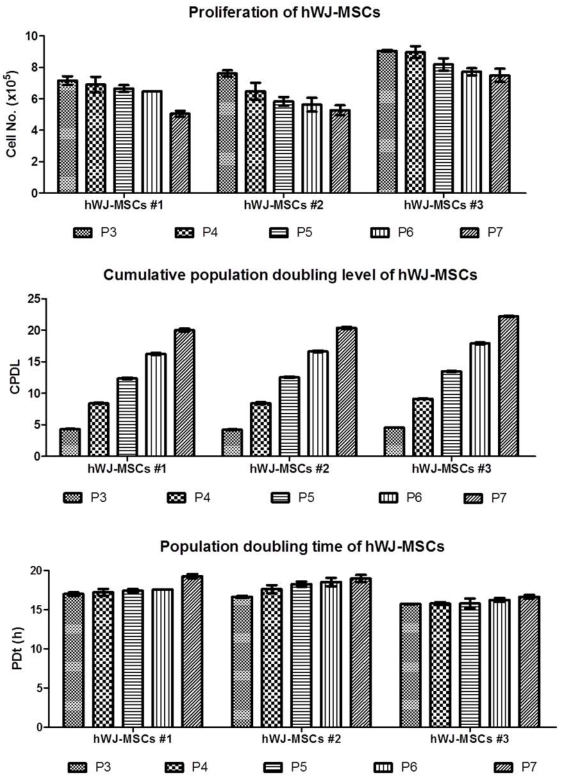

Samples	Mean ± SD				
	P3	P4	P5	P6	P7
**I. Cell number of hWJ-MSCs (×10⁵ cells)**					
hWJ-MSCs #1	7.16±0.28	6.91±0.50	6.65±0.22	6.48±0.00	5.05±0.18
hWJ-MSCs #2	7.61±0.20	6.48±0.54	5.84±0.28	5.63±0.44	5.27±0.32
hWJ-MSCs #3	9.05±0.61	8.97±0.37	8.19±0.39	7.72±0.22	7.49±0.42
**II. Cumulative population doubling level (CPDL) of hWJ-MSCs**					
hWJ-MSCs #1	4.24±0.06	8.42±0.16	12.55±0.11	16.64±0.11	20.37±0.15
hWJ-MSCs #2	4.32±0.04	8.41±0.09	12.35±0.13	16.24±0.14	20.03±0.23
hWJ-MSCs #3	4.57±0.01	9.14±0.05	13.48±0.07	17.91±0.14	22.21±0.07
**III. Population doubling time (PDt) of hWJ-MSCs**					
hWJ-MSCs #1	17.00±0.22 h	17.23±0.43 h	17.45±0.20 h	17.60±0.00 h	19.29±0.27 h
hWJ-MSCs #2	16.65±0.14 h	17.62±0.52 h	18.27±0.33 h	18.54±0.54 h	19.00±0.44 h
hWJ-MSCs #3	15.74±0.03 h	15.79±0.20 h	16.26±0.25 h	16.58±0.16 h	16.75±0.31 h

Figure S1. Growth kinetics of human Wharton's jelly–derived mesenchymal stem cells (hWJ–MSCs). Proliferation rates for various passages (P3–P7) in the different three cords were calculated by using cell number, cumulative population doubling level (CPDL), and population doubling time (PDt) analyses. Data are expressed as mean ± SD; $p < 0.05$. Data represent analysis from three independently performed experiments ($n = 3$). P = passage.

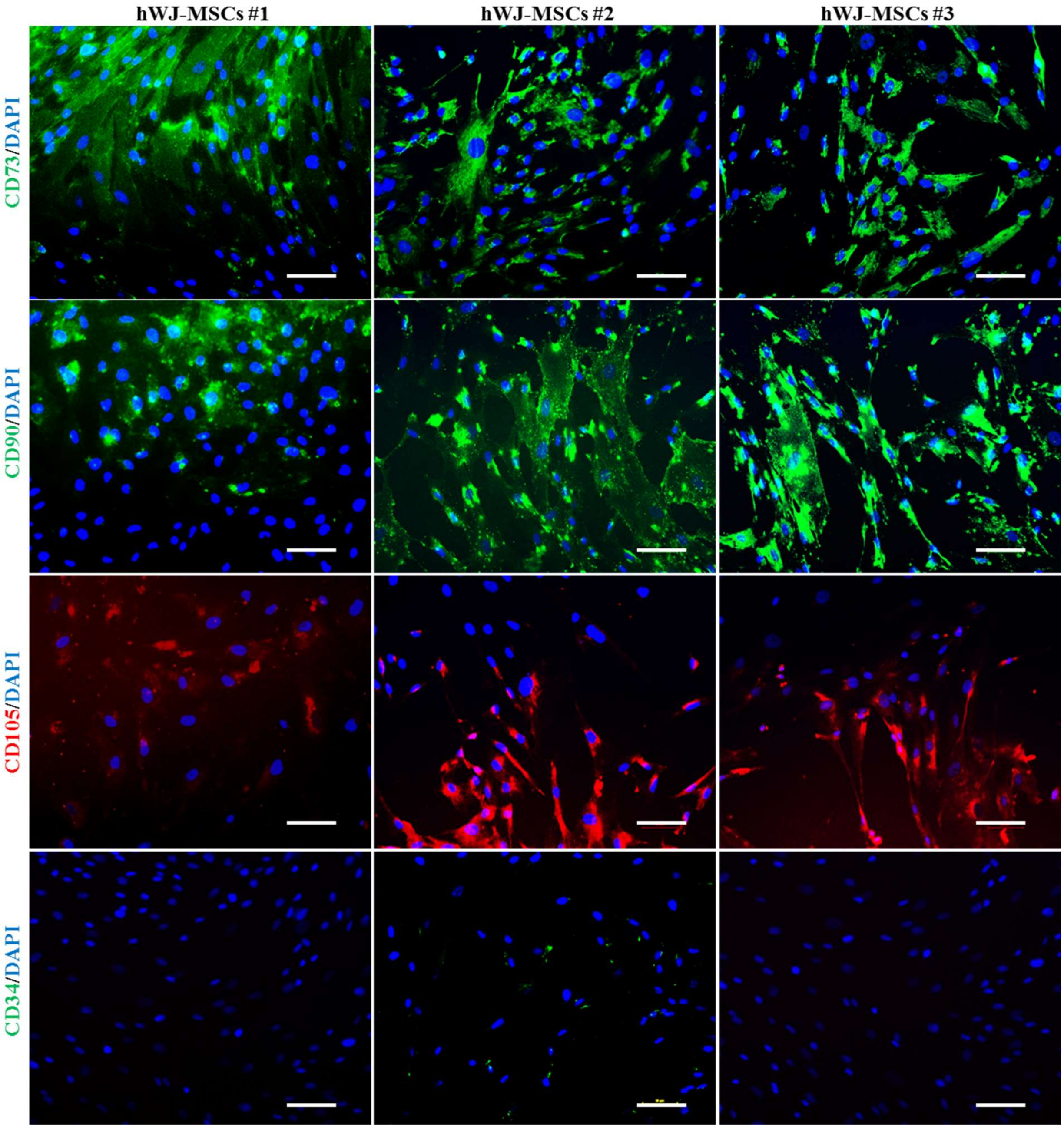

Figure S2. Characterization of human Wharton's jelly-derived mesenchymal stem cells (hWJ–MSCs) from the different three cords (*n* = 3). Representative images of the immunophenotype of hWJ–MSCs, as assessed for CD34, CD73, CD90, and CD105 staining. (Original magnifications = 200×, bar = 50 μm).

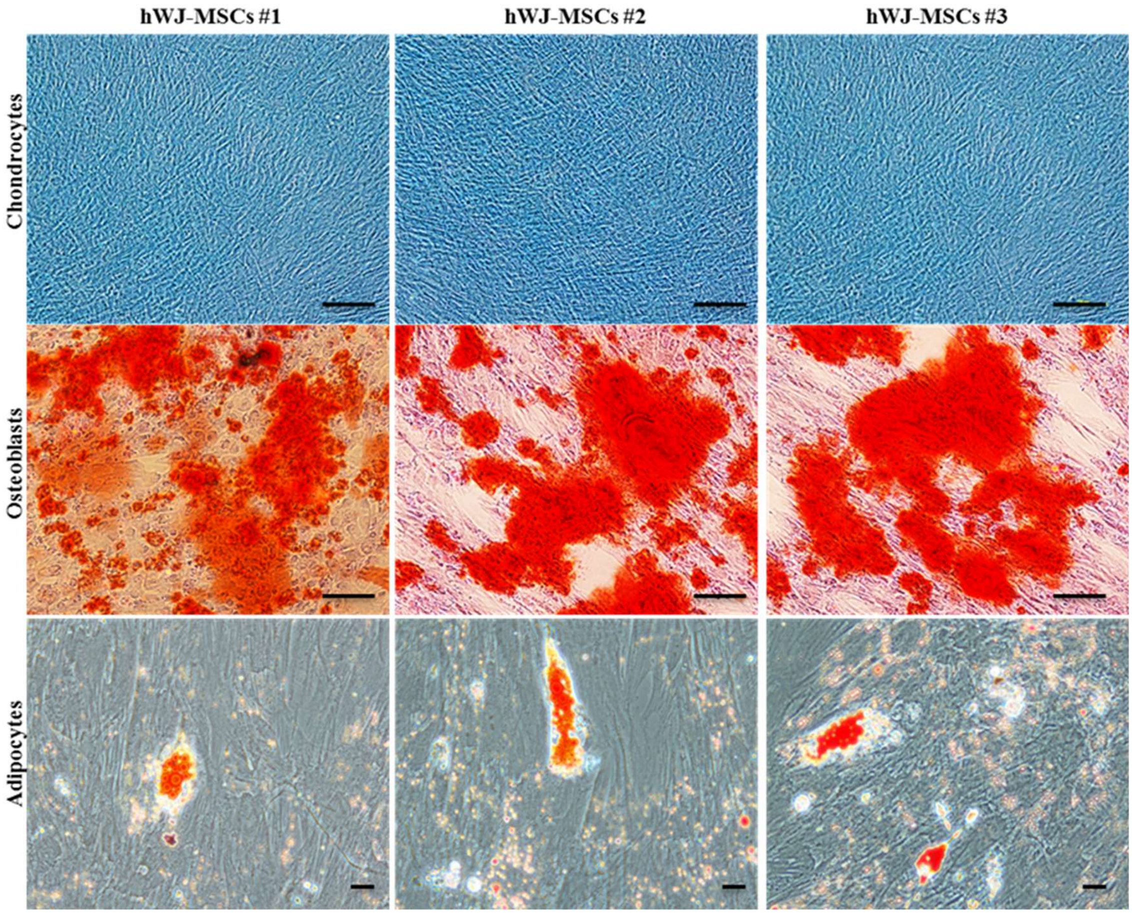

Figure S3. Characterization of human Wharton's jelly-derived mesenchymal stem cells (hWJ–MSCs) from the different three cords (n = 3). Representative images multi–lineage differentiation potential of hWJ–MSCs after 21 days, evaluated via Alcian Blue (chondrogenesis), Alizarin Red (osteogenesis), and Oil Red O (adipogenesis) staining. (Original magnifications = 100×, bar = 100 μm and 200×, bar = 50 μm).

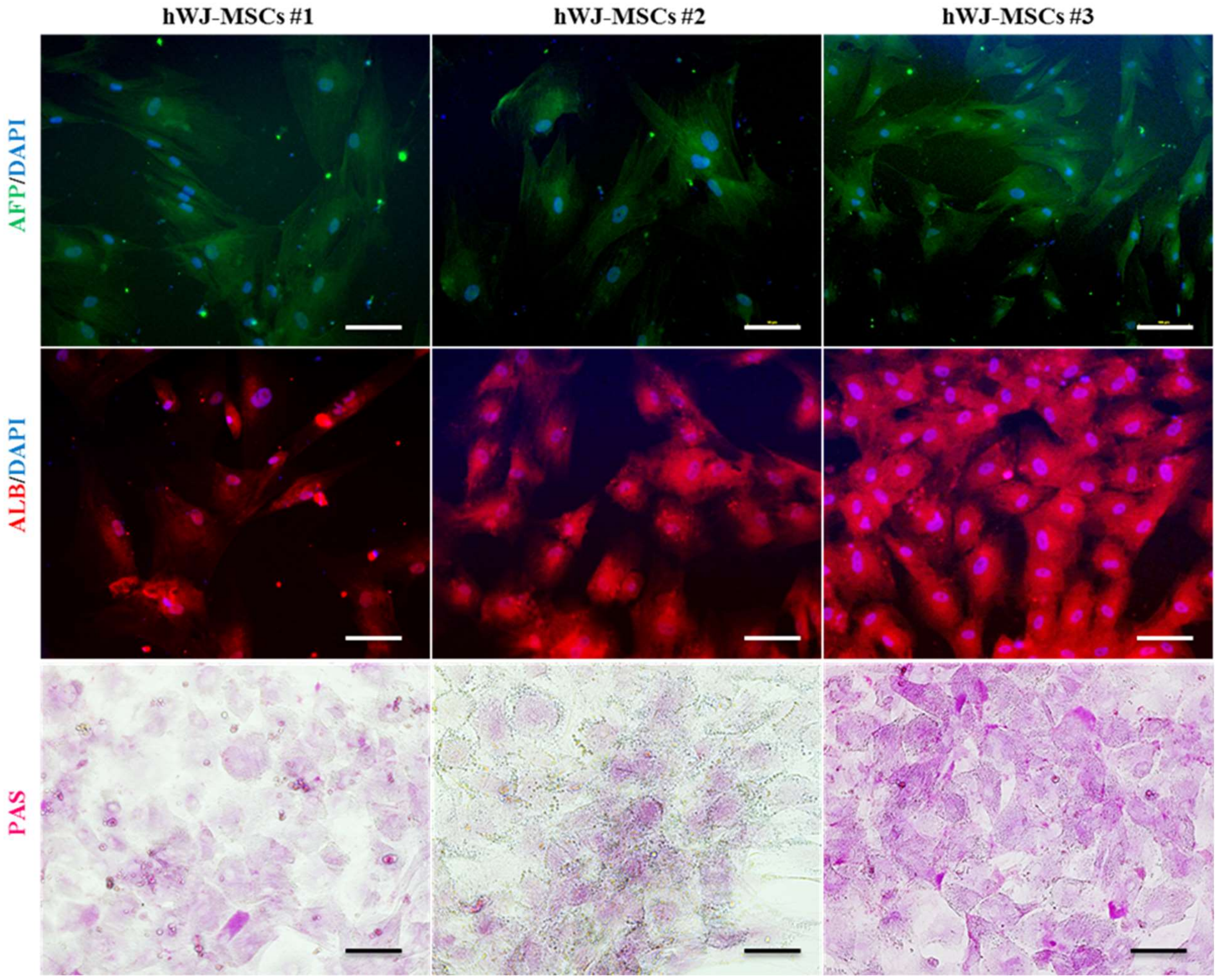

Figure S4. Hepatic protein expression and functional evaluation in differentiated human Wharton's jelly–derived mesenchymal stem cells (hWJ–MSCs; $n = 3$) with modified some standard protocol [11], assessed via immunofluorescence and Periodic acid–Schiff (PAS) staining at day 21 of differentiation. Cells were stained with a specific antibody for α-fetoprotein (AFP) and albumin (ALB). (Original magnifications 200×, bar = 50 μm).

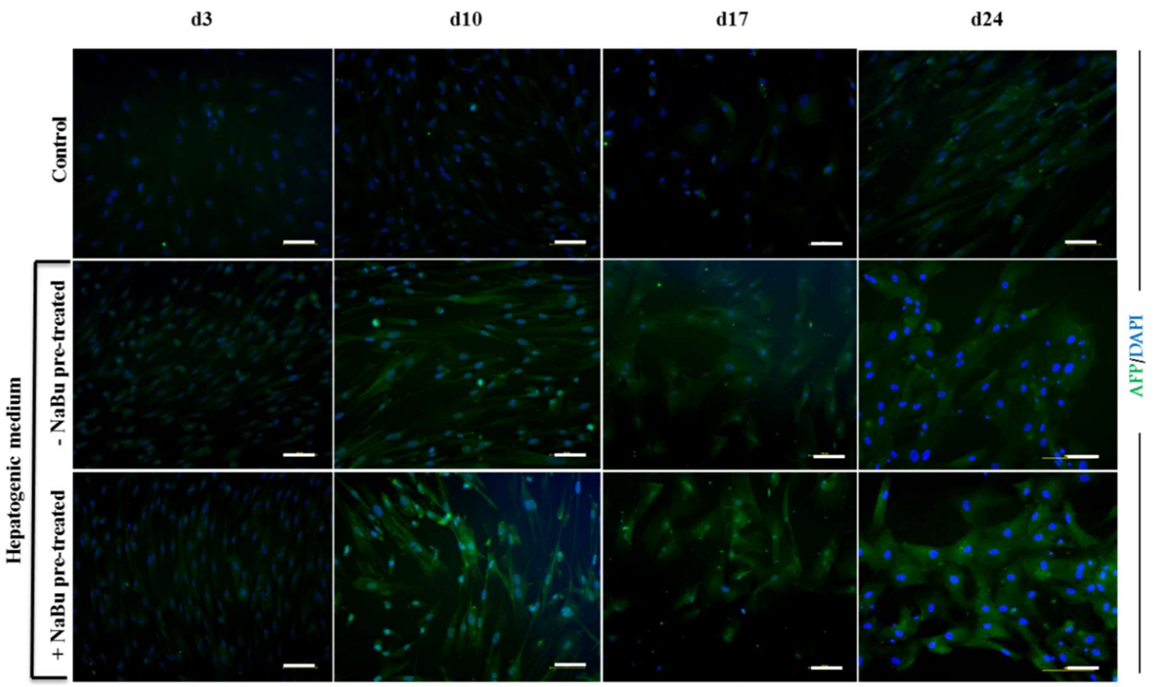

Figure S5. Hepatic protein expression in differentiated human Wharton's jelly–derived mesenchymal stem cells (hWJ–MSCs) with and without sodium butyrate (NaBu) pre-treatment and epidermal growth factor (EGF) and basic fibroblast growth factor (bFGF) supplementation, assessed via immunofluorescence analysis at days 3, 10, 17, and 24 of differentiation. The cells were stained with a specific antibody for α–fetoprotein (AFP). (Original magnifications 200×, bar = 50 μm).

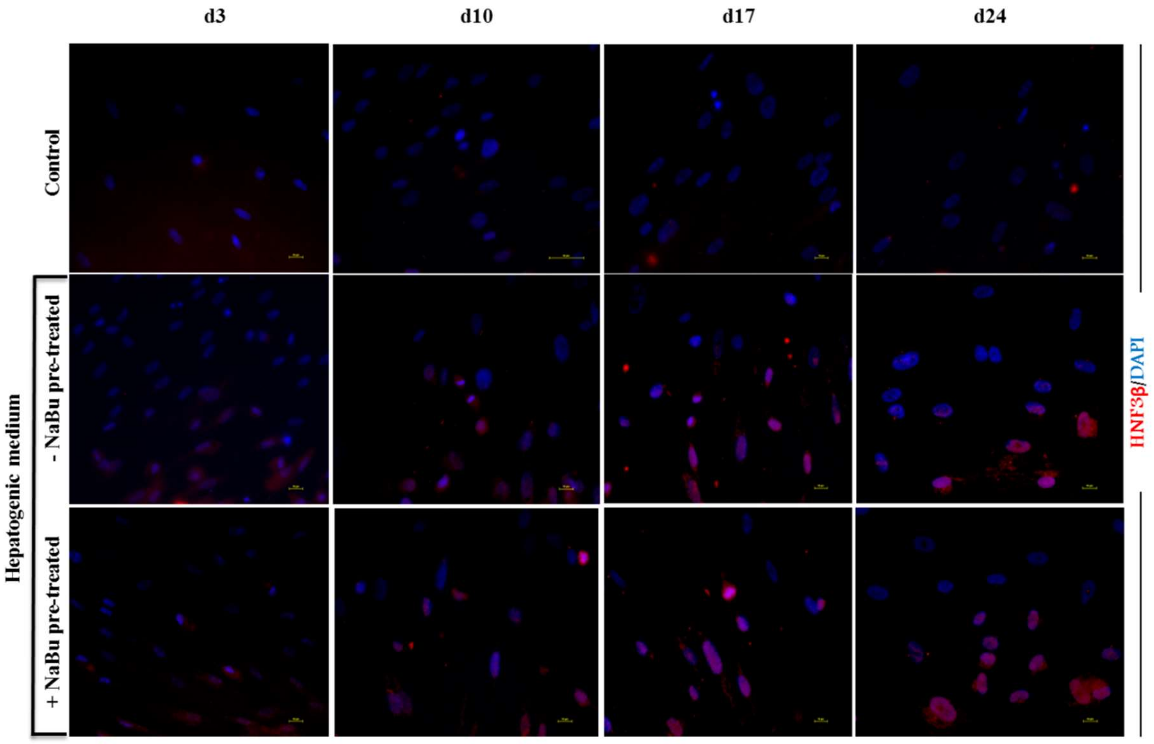

Figure S6. Hepatic protein expression in differentiated human Wharton's jelly–derived mesenchymal stem cells (hWJ–MSCs) with and without sodium butyrate (NaBu) pre-treatment and epidermal growth factor (EGF) and basic fibroblast growth factor (bFGF) supplementation, assessed via immunofluorescence analysis at days 3, 10, 17, and 24 of differentiation. The cells were stained with

a specific antibody for hepatocyte nuclear factor 3β protein (HNF3β). (Original magnifications 400×, bar = 10 μm).

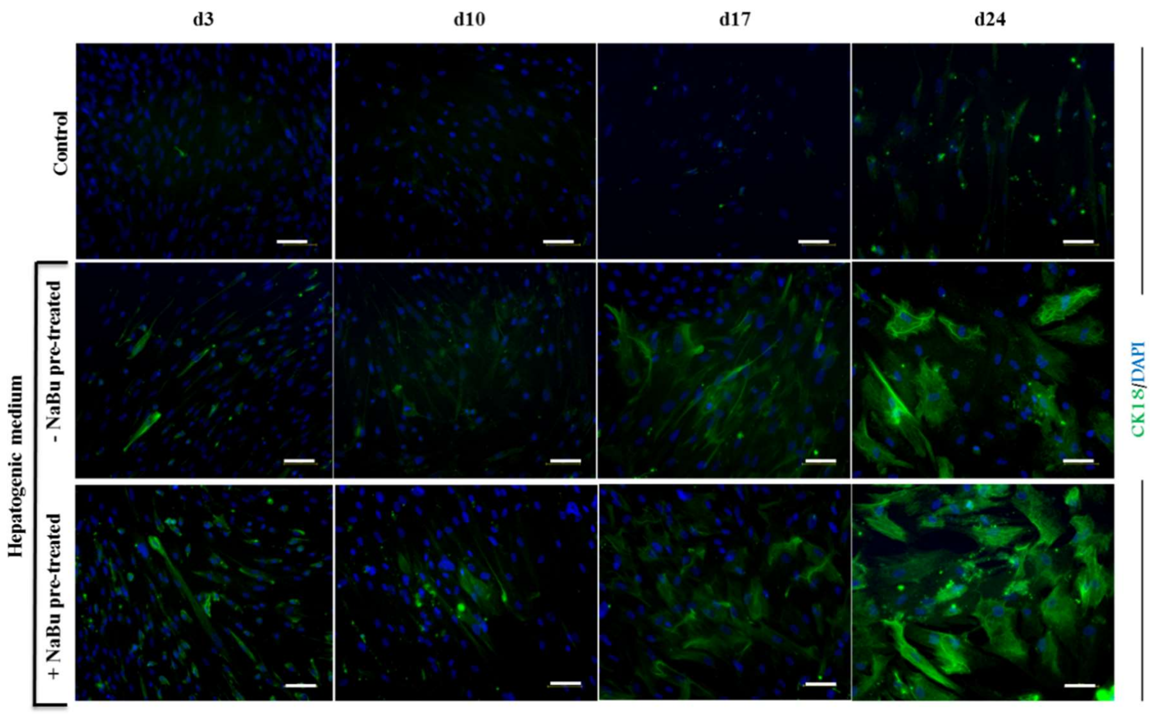

Figure S7. The hepatic protein expression in differentiated human Wharton's jelly–derived mesenchymal stem cells (hWJ–MSCs) with and without sodium butyrate (NaBu) pre-treatment and epidermal growth factor (EGF) and basic fibroblast growth factor (bFGF) supplementation assessed via immunofluorescence analysis at days 3, 10, 17, and 24 of differentiation. The cells were stained with a specific antibody for cytokeratin 18 (CK18). (Original magnifications 200×, bar = 50 μm).

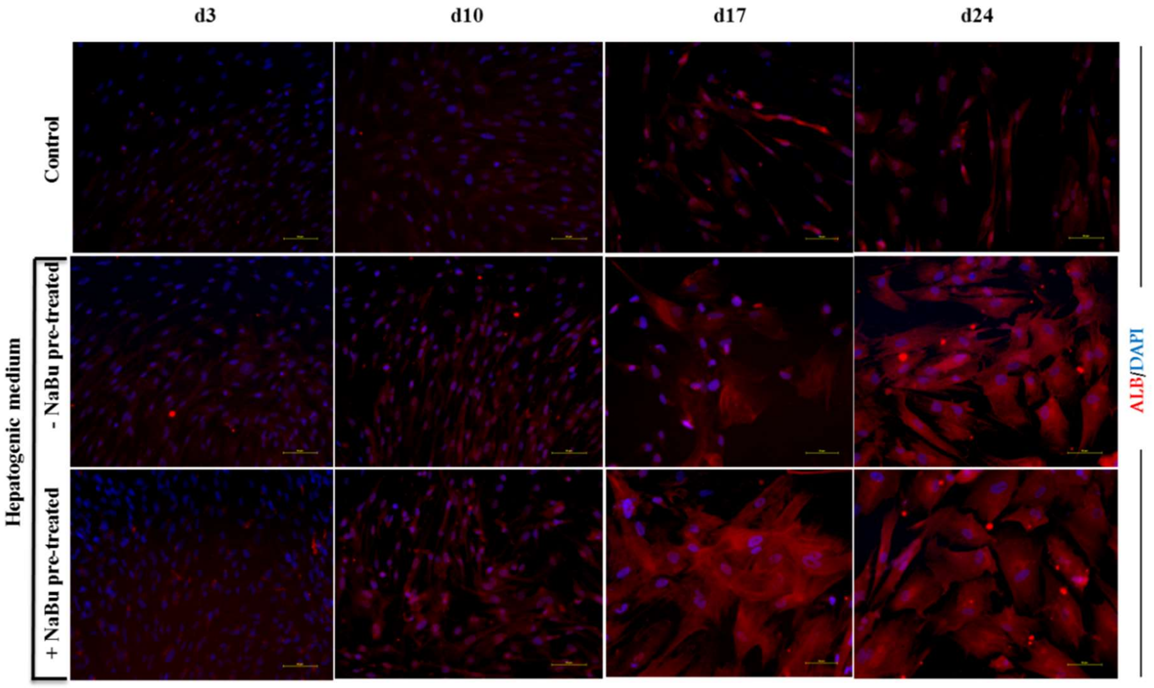

Figure S8. Hepatic protein expression in differentiated human Wharton's jelly–derived mesenchymal stem cells (hWJ–MSCs) with and without sodium butyrate (NaBu) pre-treatment and epidermal growth factor (EGF) and basic fibroblast growth factor (bFGF) supplementation assessed via

immunofluorescence analysis at days 3, 10, 17, and 24 of differentiation. The cells were stained with a specific antibody for albumin (ALB). (Original magnifications 200×, bar = 50 μm).

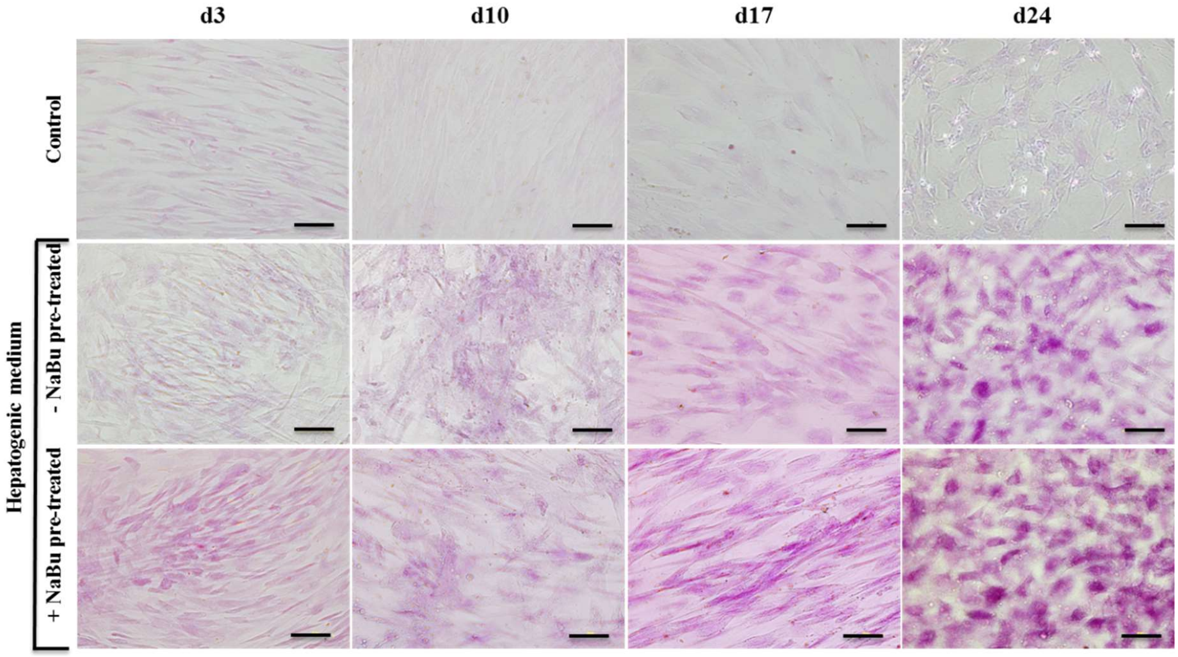

Figure S9. The functional evaluation of differentiated human Wharton's jelly–derived mesenchymal stem cells (hWJ–MSCs) with and without sodium butyrate (NaBu) pre-treatment and epidermal growth factor (EGF) and basic fibroblast growth factor (bFGF) supplementation. Glycogen storage was assessed at days 3, 10, 17, and 24 via Periodic acid-Schiff staining following the differentiation period. (Original magnifications 100×, bar = 100 μm).
